# Supplementary material for: Encystation stimuli sensing is mediated by adenylate cyclase AC2-dependent cAMP signaling in Giardia
Source: Nat Commun. 2023 Nov 9;14:7245. doi: 10.1038/s41467-023-43028-1 (PMC10636121; doi:10.1038/s41467-023-43028-1)
Supplement: Supplementary file 1 — Supplementary Information [file 41467_2023_43028_MOESM1_ESM.pdf]

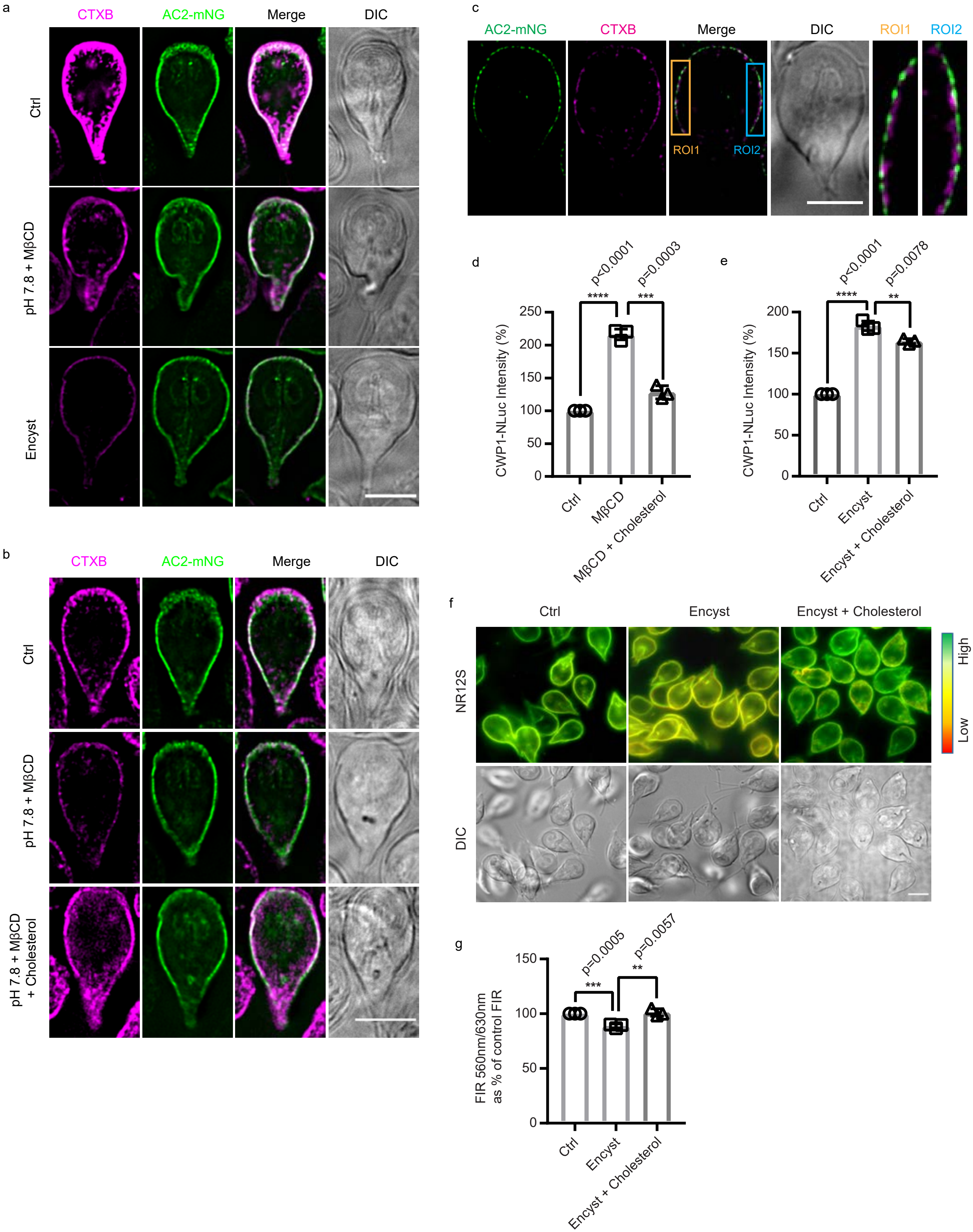

a
CTXB
AC2-mNG
Merge
DIC
Ctrl
pH 7.8 + MβCD
Encyst
b
CTXB
AC2-mNG
Merge
DIC
Ctrl
pH 7.8 + MβCD
pH 7.8 + MβCD + Cholesterol
c
AC2-mNG
CTXB
Merge
DIC
ROI1
ROI2
d
CWP1-NLuc Intensity (%)
p<0.0001
p=0.0003
****
***
0
50
100
150
200
250
Ctrl
MβCD
MβCD + Cholesterol
e
CWP1-NLuc Intensity (%)
p<0.0001
p=0.0078
****
**
0
50
100
150
200
Ctrl
Encyst
Encyst + Cholesterol
f
Ctrl
Encyst
Encyst + Cholesterol
NR12S
DIC
High
Low
g
FIR 560nm/630nm as % of control FIR
p=0.0005
p=0.0057
***
**
0
50
100
150
Ctrl
Encyst
Encyst + Cholesterol

SP Fig.1: MβCD and encystation medium altered membrane composition. ***a-b*** AC2-mNG tagged cell lines staining with 5 µg/ mL CTXB for 30 min. Cells were (***a)*** untreated, treated with 50 µM MβCD at pH 7.8, or 0.25 mg/ml porcine bile at pH 7.8., (***b***) untreated, treated with 50 µM MβCD, or 50 µM MβCD with 200 µM cholesterol at pH 7.8. The Alexa A594 images were taken with equal exposures. ***c*** Colocalization of AC2-mNG and CTXB. ***d-e*** Quantification of endogenously tagged CWP1-NLuc intensity after 4 h exposure to different encystation stimuli, including ***(d)*** 50 µM MβCD pH 7.8 only or supplemented with 200 µM cholesterol and ***(e)*** 0.125 mg/mL porcine bile pH 7.8 only or supplemented with 200 µM cholesterol. ***f*** NR12S staining of live cells. ***g*** Quantification of ratiometric NR12S fluorescent intensity of parasites incubated with 0.25mg/mL porcine bile at pH 7.8 for 1 h or the same supplemented with 200 µM cholesterol. Fluorescence was measured using a plate reader with excitation 520 nm, emission 560 nm/630 nm. Reduced fluorescence intensity ratio (FIR) 560 nm/630 nm indicates decreased plasma membrane cholesterol. Data are from three independent experiments. Scale bar, 5 µm.

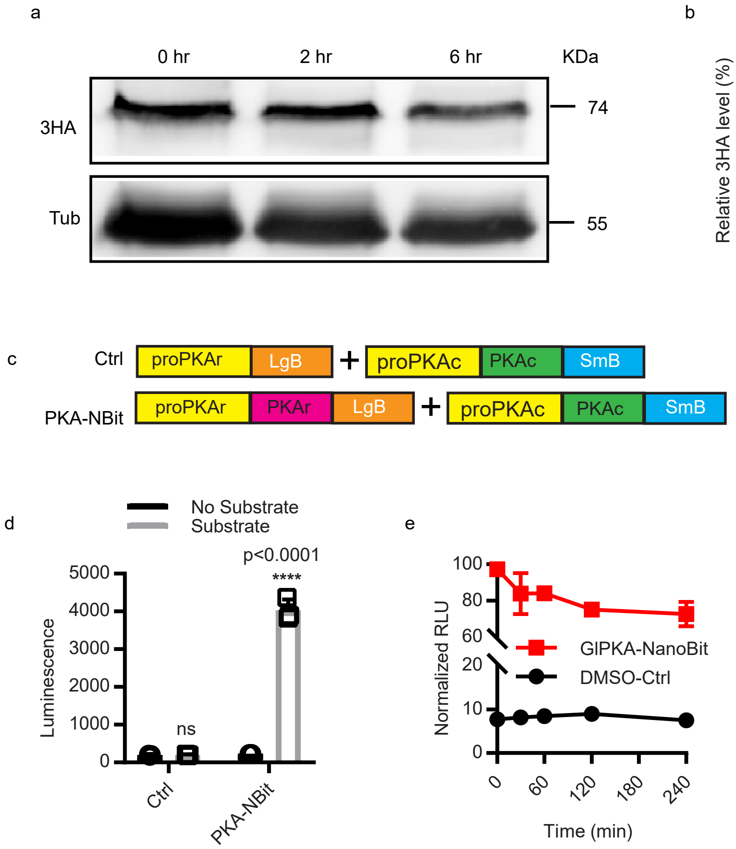

SP Fig.2: Design of GlPKA-NBit. a Western blot of PKAr-NLuc-3HA, and tubulin loading control after exposed to encystation medium for 0, 2, and 6 h. b Quantification of PKAr-NLuc-3HA normalized to tubulin. c GlPKA-NBit is composed of pPKAr::PKAr-LgB and pPKAc::PKAc-SmB, and the control is composed of pPKAr::LgB and pPKAc::PKAc-SmB. d Absolute luminescence intensity of GlPKA-NBit with or without Glo substrate. e Relative luminescence intensity of GlPKA-NBit after 0, 0.5, 1, 2, 4 h exposure to encystation medium. The data are from three biological replicates that were normalized to 0 h.

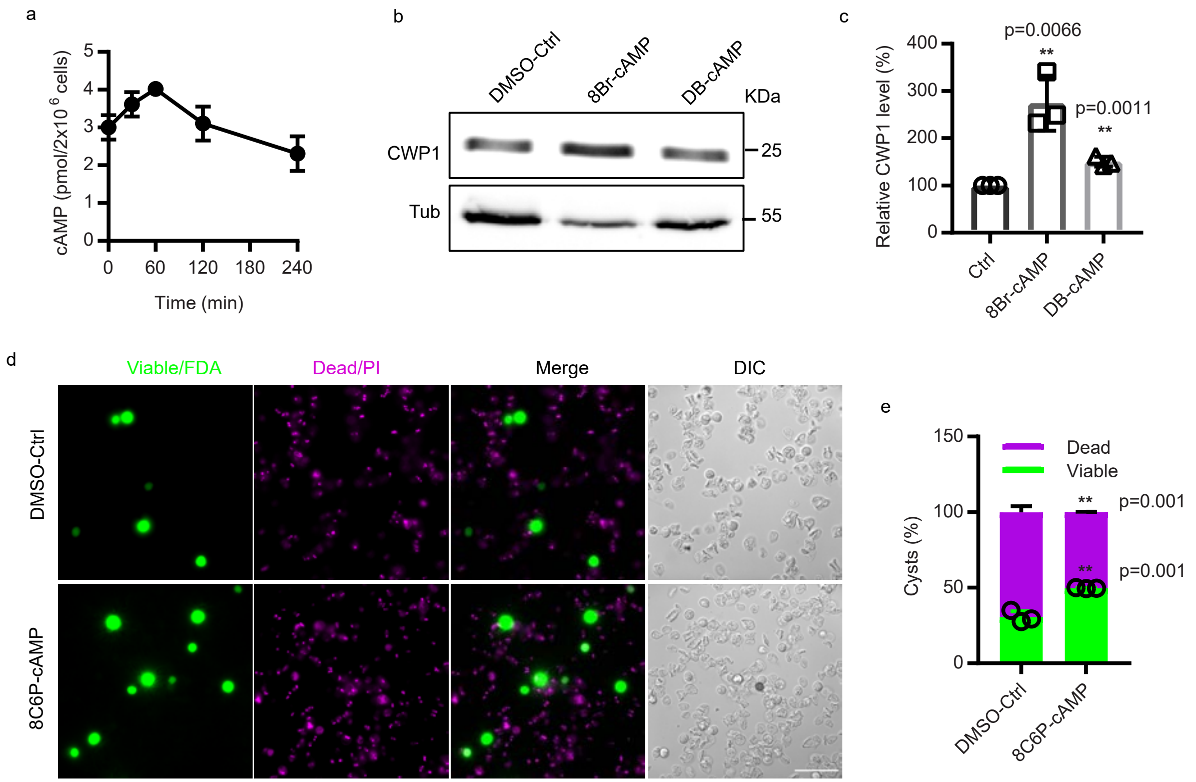

SP Fig.3: cAMP analogs enhance encystation. a Intracellular cAMP levels at 0, 0.5, 1, 2, and 4 h post encystation. 2x106 cells were collected, lysed, and measured with Caymen cAMP ELISA assay. The absorbance was detected at 405 nm using a plate reader. b Western blot of CWP1 and tubulin loading control after pretreatment with DMSO, 50μM 8Br-cAMP, or 50μM DB-cAMP. Wild type parasites were pretreated with cAMP analogs for 1h, washed with pre-encystation medium, and exposed to encystation medium. c Quantification of (b), the expression level of CWP1 is normalized to tubulin. d-e Quantification of cyst viability at 48 h post induction of encystation from parasite with or without 1 h of 8C6P-cAMP pretreatment. d Representative images of water-resistant cysts stained with fluorescein diacetate (FDA, green=live) and propidium iodine (PI, magenta=dead) after 48h exposure to encystation medium. e Quantification of viability after 1h pretreatment with 8C6P-cAMP. Total cysts counted for DMSO-Ctrl n=486, and 8C6P-cAMP n=475. Data are mean ± s.d. Scale bar, 50

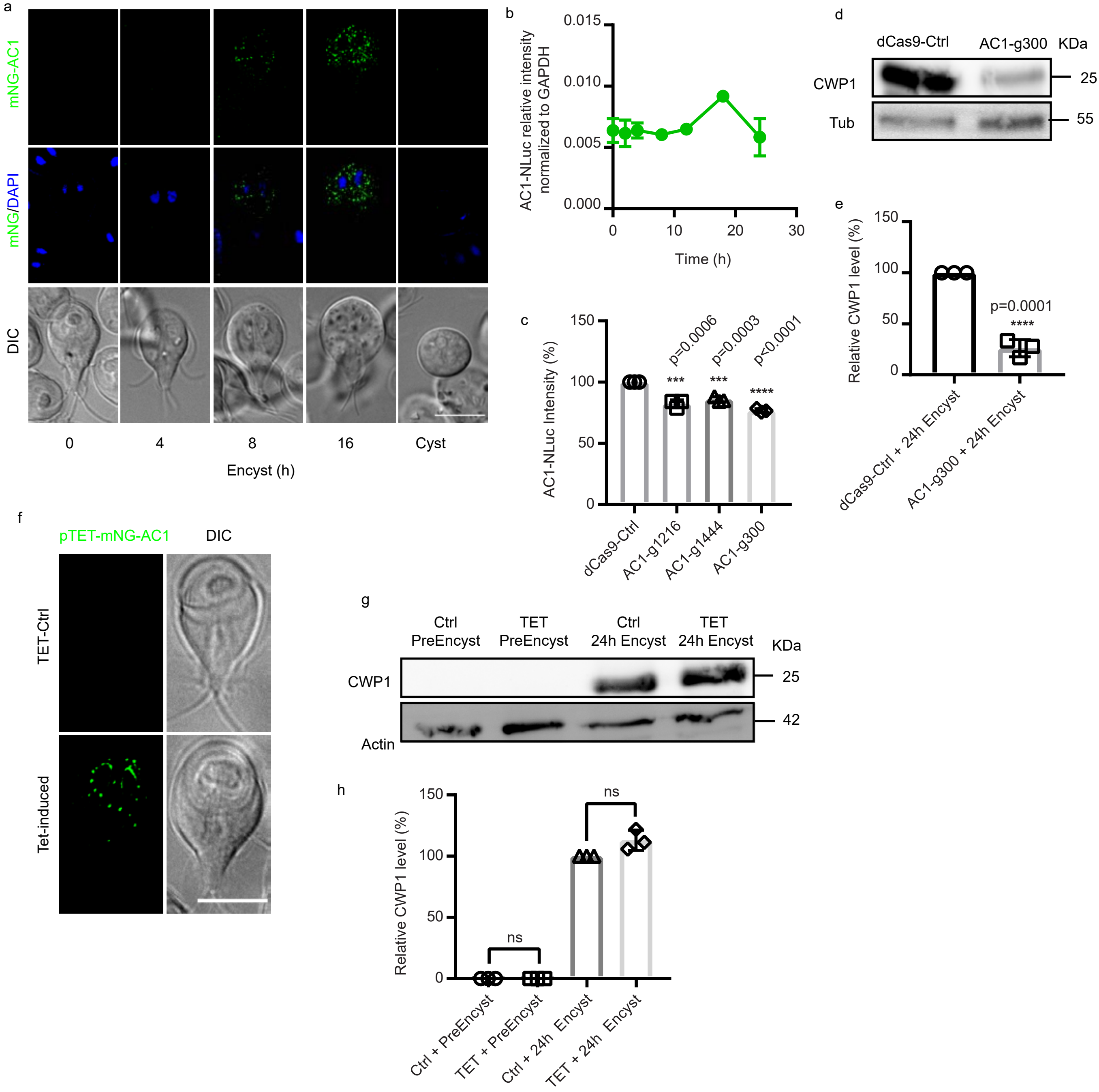

a
mNG-AC1
mNG/DAPI
DIC
0
4
8
16
Cyst
Encyst (h)
b
AC1-NLuc relative intensity normalized to GAPDH
0.015
0.010
0.005
0.000
0
10
20
30
Time (h)
c
AC1-NLuc Intensity (%)
150
100
50
0
p=0.0006
p=0.0003
p<0.0001
***
***
****
dCas9-Ctrl
AC1-g1216
AC1-g1444
AC1-g300
d
dCas9-Ctrl
AC1-g300
KDa
CWP1
Tub
25
55
e
Relative CWP1 level (%)
150
100
50
0
p=0.0001
****
dCas9-Ctrl + 24h Encyst
AC1-g300 + 24h Encyst
f
pTET-mNG-AC1
DIC
TET-Ctrl
Tet-induced
g
Ctrl PreEncyst
TET PreEncyst
Ctrl 24h Encyst
TET 24h Encyst
KDa
CWP1
Actin
25
42
h
Relative CWP1 level (%)
150
100
50
0
ns
ns
Ctrl + PreEncyst
TET + PreEncyst
Ctrl + 24h Encyst
TET + 24h Encyst

SP Fig.4: AC1 is expressed at mid-late stages of encystation. ***a*** Localization of mNG-AC1 after 0, 4, 8, 16, and 24 h of exposure to encystation stimuli. ***b*** Relative expression levels of AC1-NLuc after 0, 2, 4, 8, 12, 16, and 24 h exposures to encystation stimuli. Expression level is normalized to GAPDH::NLuc intensity. ***c*** Screening of AC1 guide RNAs. Relative AC1-NLuc levels using the indicated CRISPRi gRNAs. ***d*** Western blot of CWP1-NLuc and tubulin from dCas9 control and AC1-g300 parasites exposed to encystation medium for 24h. ***e*** Quantification of (***d***), the expression level of CWP1 is normalized using tubulin as a loading control. ***f*** Fluorescence and DIC images of pTET-AC1-mNG (AC1-mNG under the tetracycline-inducible promoter) with or without tetracycline induction. All images were taken with same exposure. (Scale bar, 10 µm). ***g*** Western blot of CWP1-NLuc and actin from pTET-AC1-mNG expressing cell lines exposed to encystation medium for 24h with or without tetracycline induction. ***h*** Quantification of (**g**), the expression level of CWP1 is normalized using actin as a loading control. 20 µg/mL tetracycline (final concentration) was added to induce expression for 24 h. Data are mean ± SD; ns, not significant. Scale bars, 5 µm.

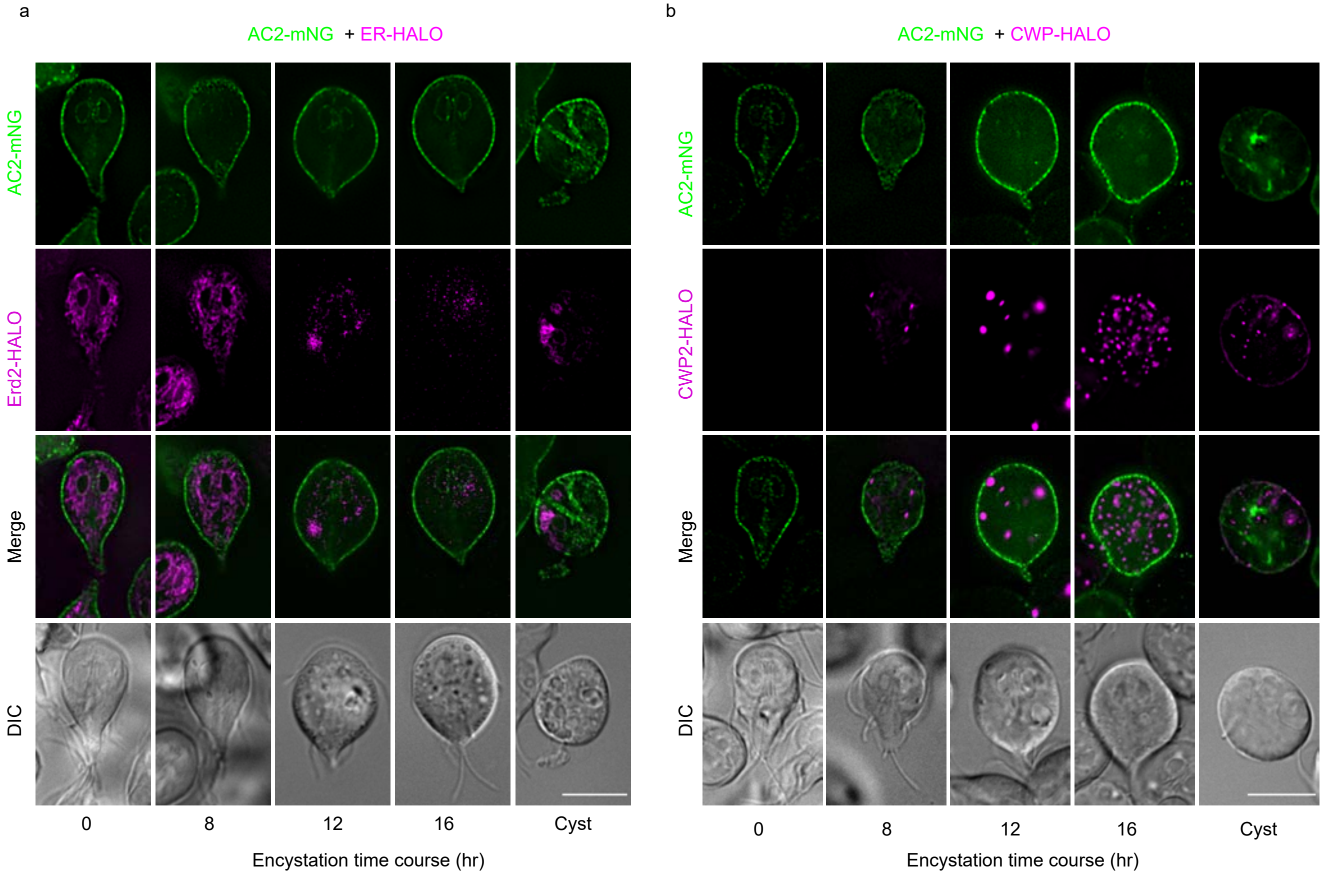

SP Fig.5: Colocalization of AC2-mNG with ER and ESV marker. a Colocalization of AC2-mNG and Erd2-Halo (ER marker) at 0, 8, 12, 16, and 24 h exposure to encystation medium. b Colocalization of AC2-mNG and CWP2-Halo (ESV marker) at 0, 8, 12, 16, and 24 h exposure to encystation medium. Scale bars, 5 μm.

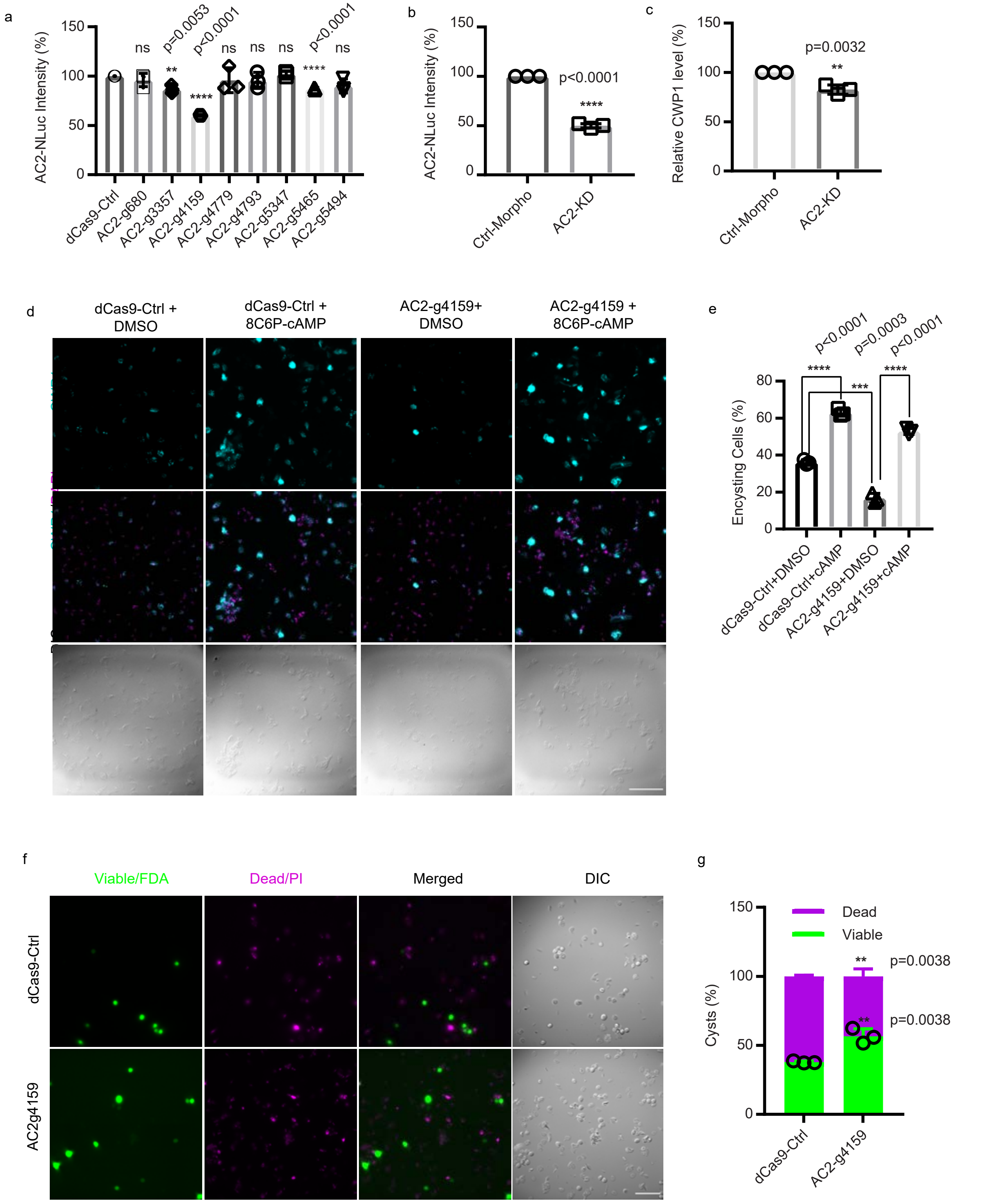
a
AC2-NLuc Intensity (%)
150
100
50
0
ns
p=0.0053
**
p<0.0001
****
ns
ns
ns
p<0.0001
****
ns
dCas9-Ctrl
AC2-g680
AC2-g3357
AC2-g4159
AC2-g4779
AC2-g4793
AC2-g5347
AC2-g5465
AC2-g5494
b
AC2-NLuc Intensity (%)
p<0.0001
****
Ctrl-Morpho
AC2-KD
c
Relative CWP1 level (%)
p=0.0032
**
Ctrl-Morpho
AC2-KD
d
dCas9-Ctrl + DMSO
dCas9-Ctrl + 8C6P-cAMP
AC2-g4159+ DMSO
AC2-g4159 + 8C6P-cAMP
e
Encysting Cells (%)
80
60
40
20
0
p<0.0001
p=0.0003
p<0.0001
****
***
****
dCas9-Ctrl+DMSO
dCas9-Ctrl+cAMP
AC2-g4159+DMSO
AC2-g4159+cAMP
f
Viable/FDA
Dead/PI
Merged
DIC
dCas9-Ctrl
AC2g4159
g
Cysts (%)
Dead
Viable
**
p=0.0038
**
p=0.0038
dCas9-Ctrl
AC2-g4159

SP Fig.6: Morpholino-mediated AC2 knockdown phenotype is consistent with CRISPRi-mediated AC2 gRNA knockdown. ***a*** Screening of AC2 guide RNAs. Relative AC2-NLuc levels using the indicated CRISPRi gRNAs. ***b***, Relative AC2-NLuc levels using morpholino-mediated AC2 knockdown. ***c*** Relative CWP1-NLuc levels using morpholino-mediated AC2 knockdown. ***d-e*** Representative images (***d***) and quantification (***e***) of 24 h encysting cells from dCas9 control and AC2-g4159 knockdown cell lines with or without 50 µM 8C6P-cAMP. Parasites were pretreated with 50 µM 8C6P-cAMP for 1h followed by 24h exposure to encystation medium (total cells counted for dCas9-Ctrl+DMSO n=1423, dCas9-Ctrl+8C6P-cAMP n=1378, AC2-g4159+DMSO n=1415, and AC2-g4159+8C6P-cAMP n=1283). ***f-g*** Representative images (***f***) and quantification (***g***) of dCas9 control and AC2-g4159 derived cysts stained with fluorescein diacetate (FDA, green=live) and propidium iodine (PI, magenta=dead). Data are mean ± s.d. from three biological replicates using student's t-test (cysts counted for dCas9-Ctrl n= 637, and AC2-g4159 n=571). Scale bars, 50 µm.

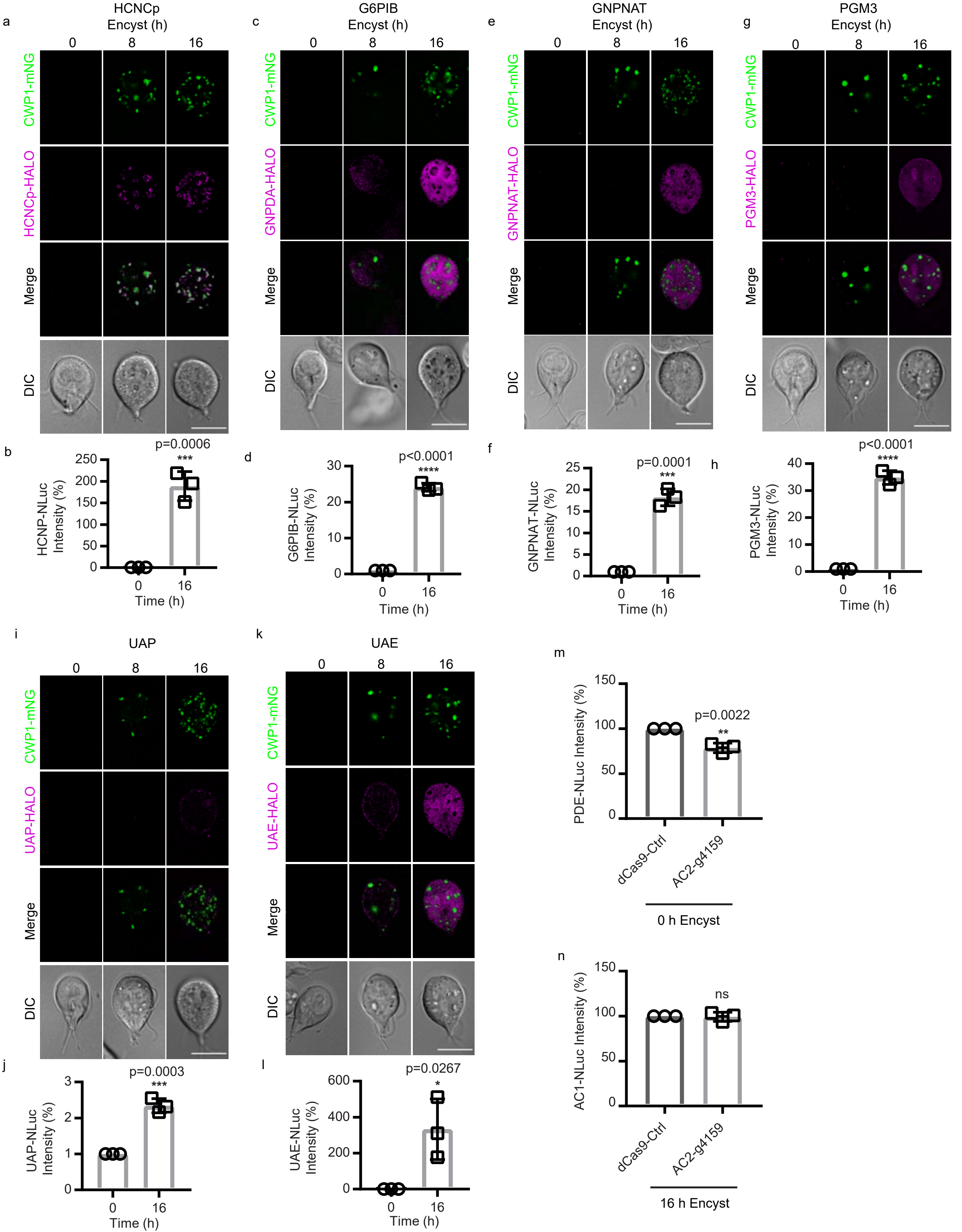

a
HCNCp
Encyst (h)
0 8 16
CWP1-mNG
HCNCp-HALO
Merge
DIC
b
p=0.0006
***
HCNP-NLuc Intensity (%)
250 200 150 100 50 0
0 16
Time (h)
c
G6PIB
Encyst (h)
0 8 16
CWP1-mNG
GNPDA-HALO
Merge
DIC
d
p<0.0001
****
G6PIB-NLuc Intensity (%)
30 20 10 0
0 16
Time (h)
e
GNPNAT
Encyst (h)
0 8 16
CWP1-mNG
GNPNAT-HALO
Merge
DIC
f
p=0.0001
***
GNPNAT-NLuc Intensity (%)
25 20 15 10 5 0
0 16
Time (h)
g
PGM3
Encyst (h)
0 8 16
CWP1-mNG
PGM3-HALO
Merge
DIC
h
p<0.0001
****
PGM3-NLuc Intensity (%)
40 30 20 10 0
0 16
Time (h)
i
UAP
0 8 16
CWP1-mNG
UAP-HALO
Merge
DIC
j
p=0.0003
***
UAP-NLuc Intensity (%)
3 2 1 0
0 16
Time (h)
k
UAE
0 8 16
CWP1-mNG
UAE-HALO
Merge
DIC
l
p=0.0267
*
UAE-NLuc Intensity (%)
600 400 200 0
0 16
Time (h)
m
PDE-NLuc Intensity (%)
150 100 50 0
p=0.0022
**
dCas9-Ctrl
AC2-g4159
0 h Encyst
n
AC1-NLuc Intensity (%)
150 100 50 0
ns
dCas9-Ctrl
AC2-g4159
16 h Encyst

SP Fig.7: GalNAc biosynthesis enzymes are upregulated at 16h post induction of encystation. ***a-l*** Relative expression levels of HCNCp and GalNAc biosynthesis enzymes (***a***) HCNCp-Halo localization and (***b***) HCNCp-NLuc (GL50803_40376) intensity, (***c***) G6PI-B-Halo localization and (***d***) G6PI-B-NLuc (GL50803_8245) intensity, (***e***) GNPNAT-Halo localization and (***f***) GNPNAT-NLuc (GL50803_14259) intensity, (***g***) PGM3-Halo localization and (***h***) PGM3-NLuc (GL50803_16069) intensity, (***i***) UAP-Halo localization and (**j**) UAP-NLuc (GL50803_16217) intensity, (***k***) UAE-Halo localization and (***l***) UAE-NLuc (GL50803_7982) intensity. HCNCp and GalNAc biosynthesis enzymes were tagged with Halo and labeled with Halo tag ligand JF646. CWP1 was tagged with mNeonGreen. Halo tagged cell lines were imaged at 0, 8, 16 h post exposure to encystation medium. All images in were taken with equal exposure. The luminescence of NLuc tagged cell lines was measured at 16 h post induction of encystation using a plate reader. Data are mean ± s.d. from three biological replicates using student's t-test. Scale bars, 5 µm. ***m-n*** Relative expression of PDE-NLuc at 0 h and AC1-NLuc at 24h post exposure to encystation medium from dCas9-Ctrl and AC2-g4159 knockdown. Data are mean ± SD; ns, not significant.

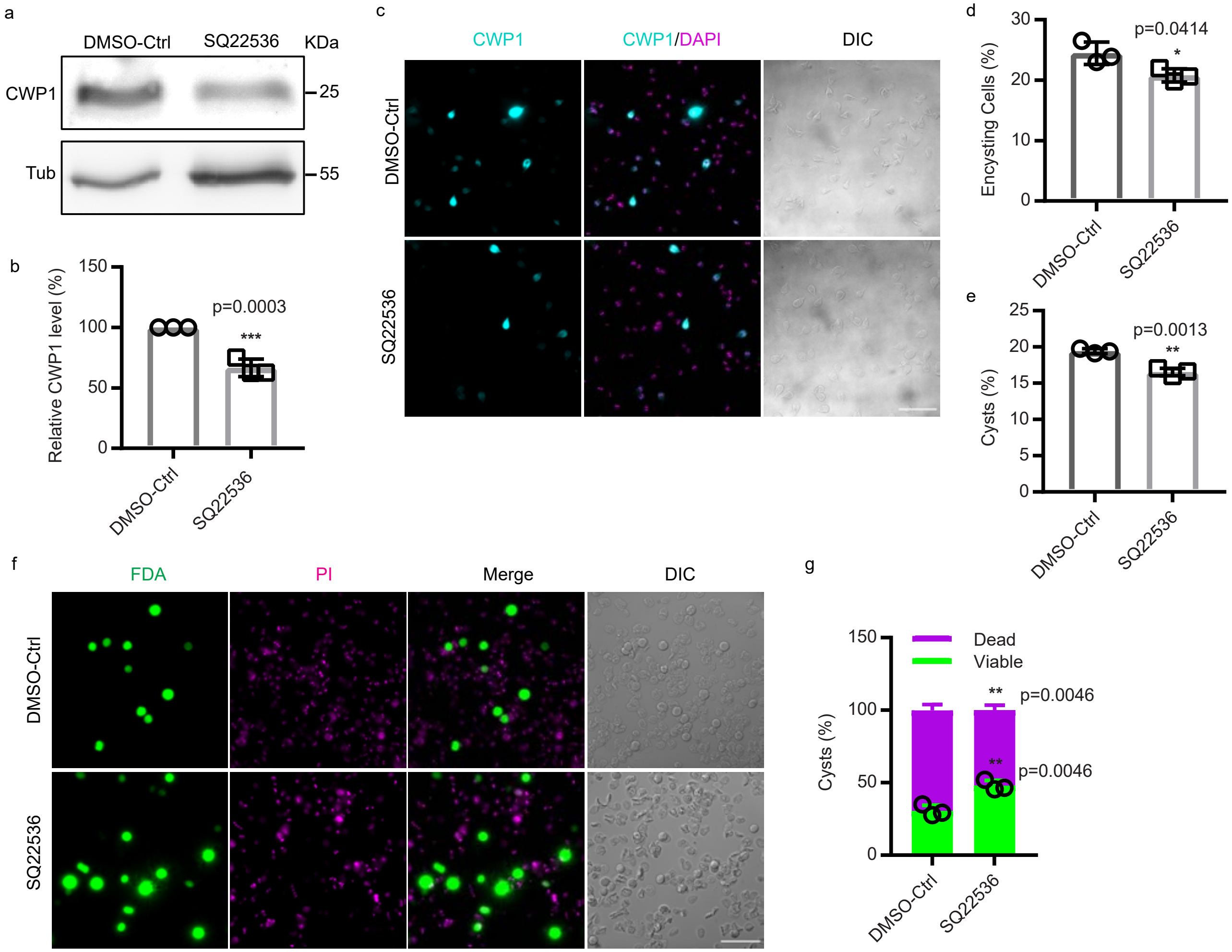

SP Fig.8: AC inhibitor SQ22536 impairs encystation. a Western blot of CWP1 and tubulin from 1 h pretreatment of DMSO control and SQ22536 at 4h post exposure to encystation medium. b Quantification of (a), CWP1 normalized to tubulin. c-d Representative images (c) and quantification (d) of 24 h encysting cells from DMSO control and SQ22536 treated parasite. Parasites were pretreated with 10 μM SQ22536 for 1 h followed by 24 h exposure to encystation medium (total cells counted for DMSO-Ctrl n= 1002, and SQ22536 n=1028). e Quantification of mature cysts at 48 h post induction of encystation from parasites pretreated with DMSO or SQ22536 for 1 h. Cyst counts were performed by hemocytometer (total cells counted for DMSO-Ctrl n=1730, and SQ22536 n=1463). Data are mean ± s.d. f-g Representative images (f) and quantification (g) of DMSO control and SQ22536-treated cysts stained with fluorescein diacetate (FDA, green=live) and propidium iodine (PI, magenta=dead). Data are mean ± s.d. from three biological replicates using student's t-test (cysts counted for dCas9-ctrl n=486, and SQ22536 n=441). Scale bars, 50 μm.

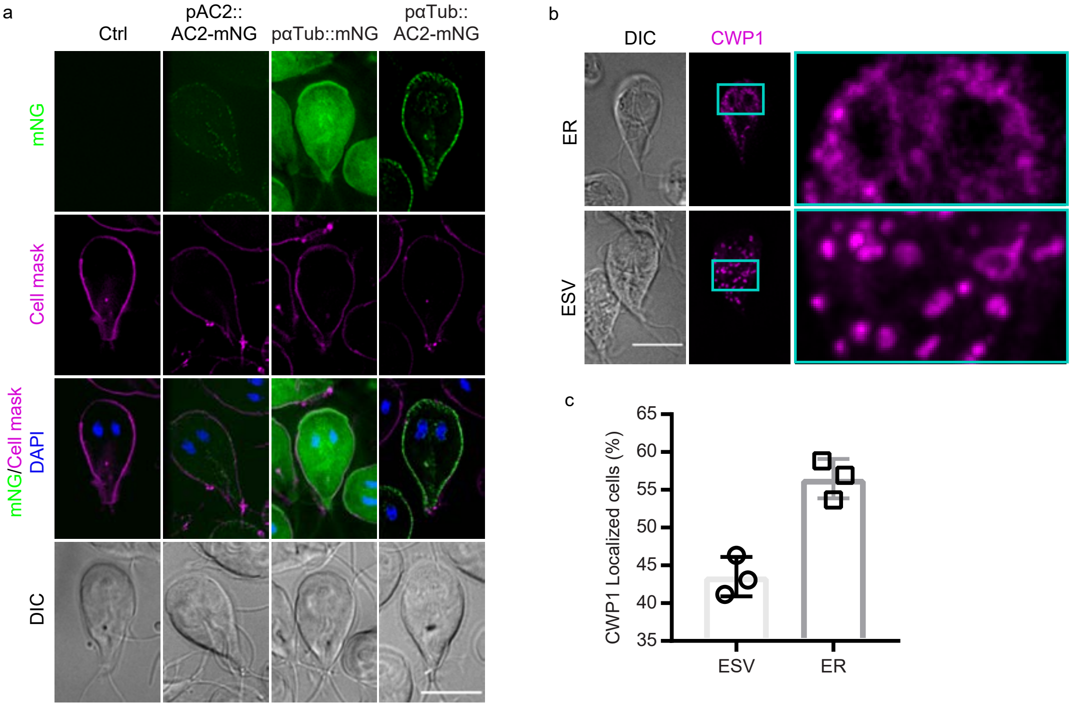

SP Fig.9: AC2 overexpression initiates encystation. a Representative images of mNeonGreen from the indicated cell lines. The images were taken using equal exposure. b Representative CWP1 IFA images and (b) categorization of CWP1 localization to the ER or ESVs. Data are mean ± s.d. from three biological replicates using student's t-test (total cell counted n=239). Scale bars, 5 µm.
