# Supplementary material for: Encystation stimuli sensing is mediated by adenylate cyclase AC2-dependent cAMP signaling in Giardia
Source: Nat Commun. 2023 Nov 9;14:7245. doi: 10.1038/s41467-023-43028-1 (PMC10636121; doi:10.1038/s41467-023-43028-1)
Supplement: Supplementary file 3 — Description of Additional Supplementary Files [file 41467_2023_43028_MOESM3_ESM.docx]

**Description of Additional Supplementary Files**

**Title:** Supplementary data 1

**Description:** Primers used in the present study, restriction enzymes used for cloning, and linearization of integrated constructs
