# Supplementary material for: Encystation stimuli sensing is mediated by adenylate cyclase AC2-dependent cAMP signaling in Giardia
Source: Nat Commun. 2023 Nov 9;14:7245. doi: 10.1038/s41467-023-43028-1 (PMC10636121; doi:10.1038/s41467-023-43028-1)
Supplement: Supplementary file 5 — Reporting Summary [file 41467_2023_43028_MOESM5_ESM.pdf]

nature portfolio

Corresponding author(s): Alexander Paredez

Last updated by author(s): 10/19/2023

Reporting Summary

Nature Portfolio wishes to improve the reproducibility of the work that we publish. This form provides structure for consistency and transparency in reporting. For further information on Nature Portfolio policies, see our Editorial Policies and the Editorial Policy Checklist.

Statistics

For all statistical analyses, confirm that the following items are present in the figure legend, table legend, main text, or Methods section.

n/a	Confirmed	
☐	☒	The exact sample size (n) for each experimental group/condition, given as a discrete number and unit of measurement
☐	☒	A statement on whether measurements were taken from distinct samples or whether the same sample was measured repeatedly
☐	☒	The statistical test(s) used AND whether they are one- or two-sided<br>*Only common tests should be described solely by name; describe more complex techniques in the Methods section.*
☐	☒	A description of all covariates tested
☒	☐	A description of any assumptions or corrections, such as tests of normality and adjustment for multiple comparisons
☐	☒	A full description of the statistical parameters including central tendency (e.g. means) or other basic estimates (e.g. regression coefficient) AND variation (e.g. standard deviation) or associated estimates of uncertainty (e.g. confidence intervals)
☐	☒	For null hypothesis testing, the test statistic (e.g. F, t, r) with confidence intervals, effect sizes, degrees of freedom and P value noted<br>*Give P values as exact values whenever suitable.*
☒	☐	For Bayesian analysis, information on the choice of priors and Markov chain Monte Carlo settings
☒	☐	For hierarchical and complex designs, identification of the appropriate level for tests and full reporting of outcomes
☒	☐	Estimates of effect sizes (e.g. Cohen's d, Pearson's r), indicating how they were calculated

Our web collection on statistics for biologists contains articles on many of the points above.

Software and code

Policy information about availability of computer code

Data collection	Softworx 7.0.0 GE Healthcare was used for microscopy image collection and deconvolution, Image Lab (BioRad) was used for western blot acquisition, SparkControl 3.0 software was used for fluorescent plate reader and luminescence measurements.
Data analysis	Fiji/ImageJ 2.15.0 was used for image analysis and western blot densitometry.

For manuscripts utilizing custom algorithms or software that are central to the research but not yet described in published literature, software must be made available to editors and reviewers. We strongly encourage code deposition in a community repository (e.g. GitHub). See the Nature Portfolio guidelines for submitting code & software for further information.

Data

Policy information about availability of data

All manuscripts must include a data availability statement. This statement should provide the following information, where applicable:

- Accession codes, unique identifiers, or web links for publicly available datasets
- A description of any restrictions on data availability
- For clinical datasets or third party data, please ensure that the statement adheres to our policy

Data supporting the findings of this work are available within the article and its Supplementary Information files. Raw values used for graphs in the figures are in Source Data and uncropped blots are in Supplementary Data2. All Giardia DNA sequences used in this study are available at giardiadb.org.

Research involving human participants, their data, or biological material

Policy information about studies with human participants or human data. See also policy information about sex, gender (identity/presentation), and sexual orientation and race, ethnicity and racism.

Reporting on sex and gender	NA
Reporting on race, ethnicity, or other socially relevant groupings	NA
Population characteristics	NA
Recruitment	NA
Ethics oversight	NA

Note that full information on the approval of the study protocol must also be provided in the manuscript.

Field-specific reporting

Please select the one below that is the best fit for your research. If you are not sure, read the appropriate sections before making your selection.

[x] Life sciences   [ ] Behavioural & social sciences   [ ] Ecological, evolutionary & environmental sciences

For a reference copy of the document with all sections, see nature.com/documents/nr-reporting-summary-flat.pdf

Life sciences study design

All studies must disclose on these points even when the disclosure is negative.

Sample size	No power calculations were made. Since we have an abundance of cells in each experiment we aimed to count 1000 cells over three biological replicates. This is far more than in some studies and since we were able to identify statistically significant differences we are confident we exceeded the minimum necessary cell count. For other experiments such as plate reader assays or western blots we performed three biological replicates as is standard practice for most studies, each sample includes thousands of cells to produce robust signals so these experiments average population level responses. Again statistically significant differences were identified so we are confident that the n of our experiments is sufficient.
Data exclusions	No data was excluded except for some entire western blots that had excessive degradation and were not interpretable.
Replication	We used three biologically independent replicates for each experiment. Samples came from different culture tubes on different days. With the exception of western blots all replicates were successful. Some western blots had excessive degradation or loading problems and had to be repeated.
Randomization	Samples were not randomized, but we were careful to treat all samples identically in terms of exposure times, cells numbers in each sample etc. with the goal of unbiased data acquisition.
Blinding	First Author Han-Wei Shih typically performed initial experiments in an unblinded manner, but then would hand off blinded samples to second author Germain Alas to test reproducibility. For plate reader experiments or western blots no blinding was performed since the analysis is strictly quantitative. and cells were diluted to identical concentrations after counting density with a MoxiZ Coulter counter.

Reporting for specific materials, systems and methods

We require information from authors about some types of materials, experimental systems and methods used in many studies. Here, indicate whether each material, system or method listed is relevant to your study. If you are not sure if a list item applies to your research, read the appropriate section before selecting a response.

Materials & experimental systems

n/a	Involved in the study	
[ ]	[x]	Antibodies
[ ]	[x]	Eukaryotic cell lines
[x]	[ ]	Palaeontology and archaeology
[x]	[ ]	Animals and other organisms
[x]	[ ]	Clinical data
[x]	[ ]	Dual use research of concern
[x]	[ ]	Plants

Methods

n/a	Involved in the study	
[x]	[ ]	ChIP-seq
[x]	[ ]	Flow cytometry
[x]	[ ]	MRI-based neuroimaging

Antibodies

Antibodies used	6-11B-1 anti-tubulin (Sigma T7451), 28 PB+1 rabbit anti-GiardiaActin (custom Paredez 2011), A300 Anti-CWP1 (Waterborne), anti-IgG2b mouse isotype-specific antibody conjugated with Alexa 488 (Molecular Probes A-21141)
Validation	6-11B-1 PMID: 32459558, 28 PB+1 PMID: 21444821, A300 is a commercial anti-CWP1 antibody used in many pubs including PMID: 12711599.

Eukaryotic cell lines

Policy information about cell lines and Sex and Gender in Research

Cell line source(s)	Parental cell line WB C6 was originally acquired from the ATCC #50803. Transgenic cell lines derived from this were generated by electroporating in constructs that had been sequenced.
Authentication	Episomal cell lines were not validated beyond confirming antibiotic resistance and expression of a tagged protein, for integrated cell lines we did not include a promoter so any observed mNeonGreen or NanoLuc expression could only result from a successful integration event.
Mycoplasma contamination	cell lines were not tested, Mycoplasm is not a known issue for Giardia lamblia culture
Commonly misidentified lines (See ICLAC register)	NA
