# Supplementary material for: Encystation stimuli sensing is mediated by adenylate cyclase AC2-dependent cAMP signaling in Giardia
Source: Nat Commun. 2023 Nov 9;14:7245. doi: 10.1038/s41467-023-43028-1 (PMC10636121; doi:10.1038/s41467-023-43028-1)

Fig4a_Western blot_8C6P-cAMP

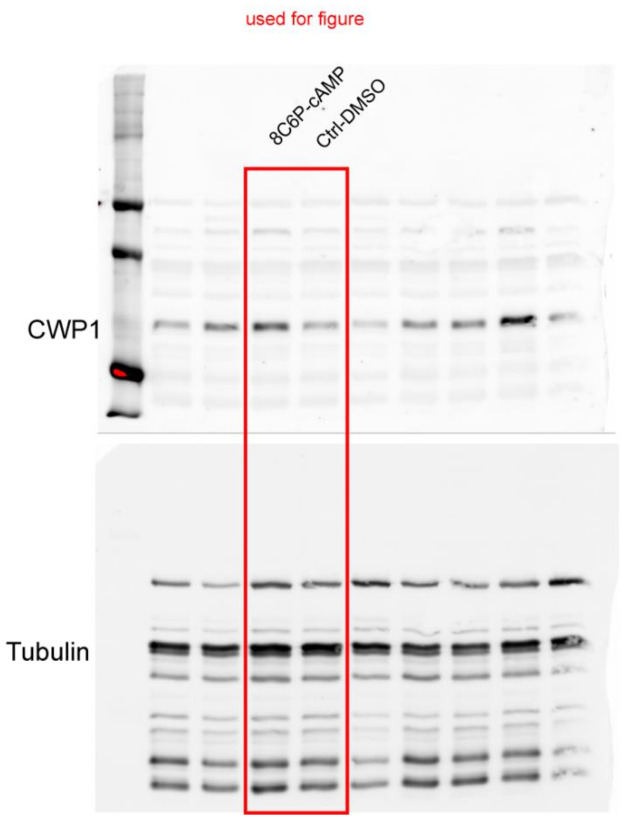

Fig5f Western blot: AC2g4159 Encysted

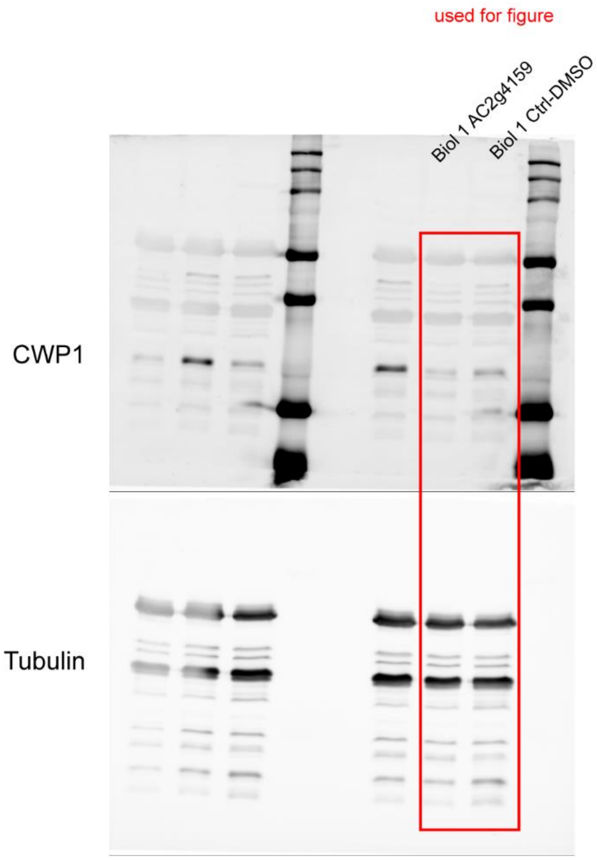

Fig7d Western blot:  pαTub::mNG and pαTub::AC2-mNG

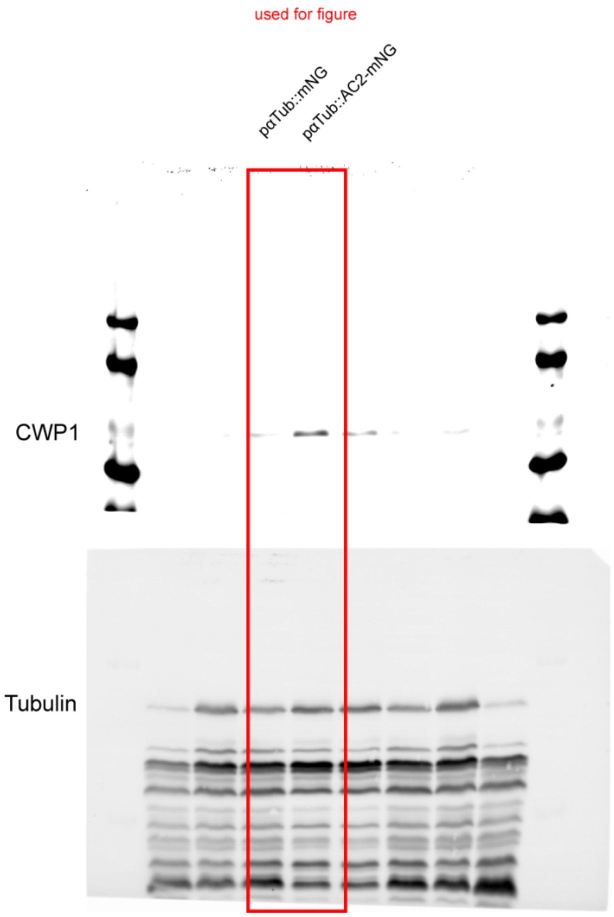

Supplementary Fig2a Western blot: PKAr-Nluc-3HA Encysted

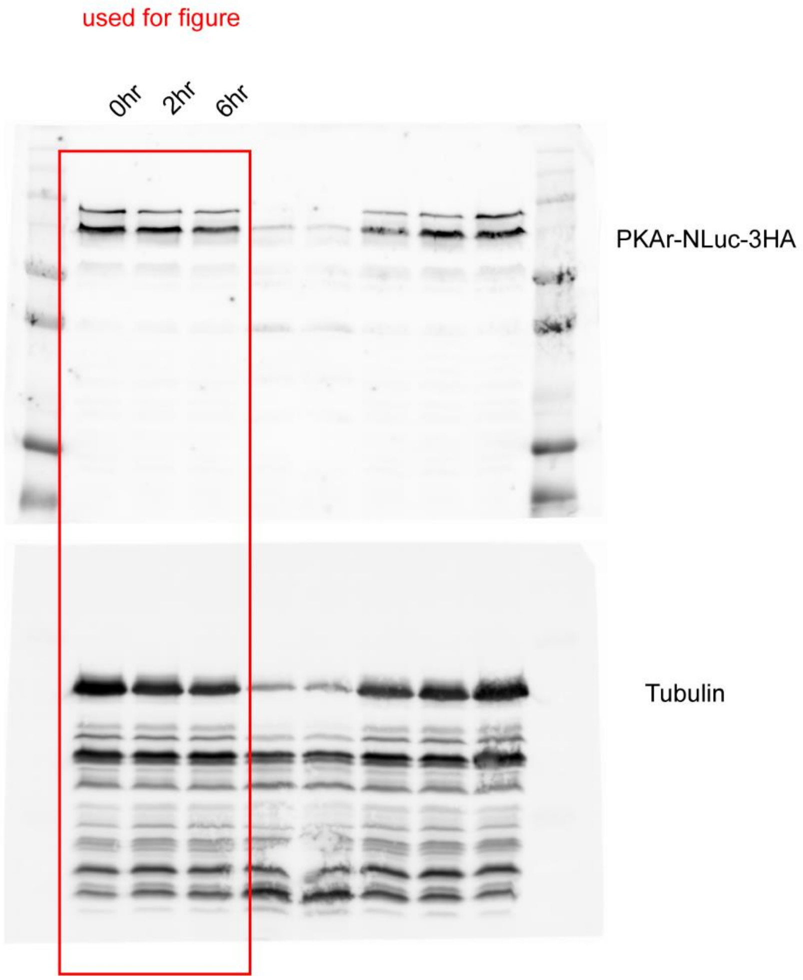

Supplementary Fig3b Western blot: cAMP analogs 8Br-cAMP and DB-cAMP Encysted

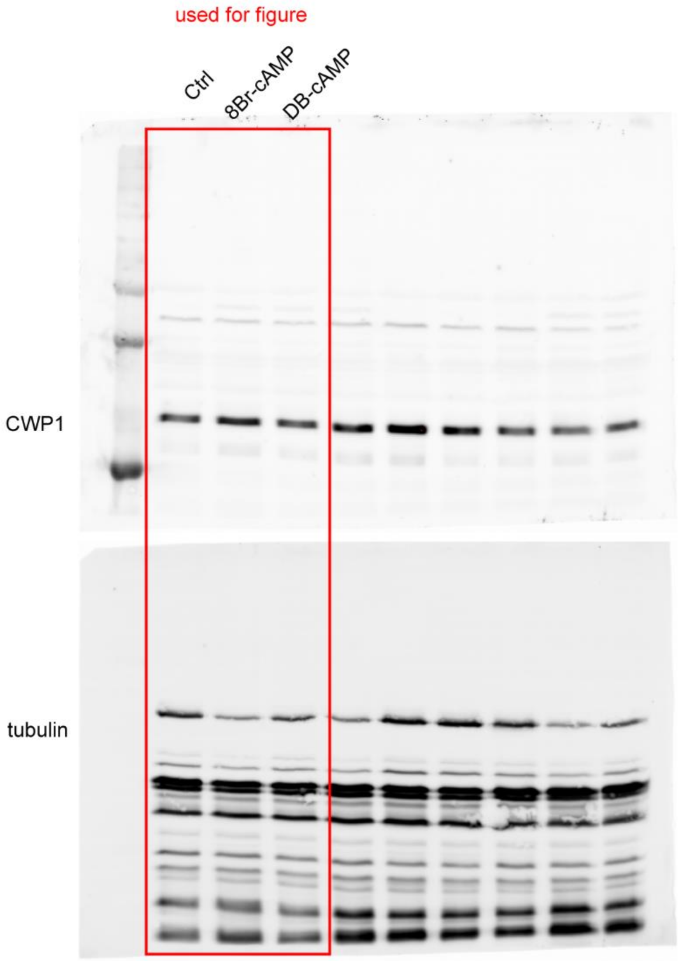

Supplementary Fig4d Western blot: AC1g300 Encysted

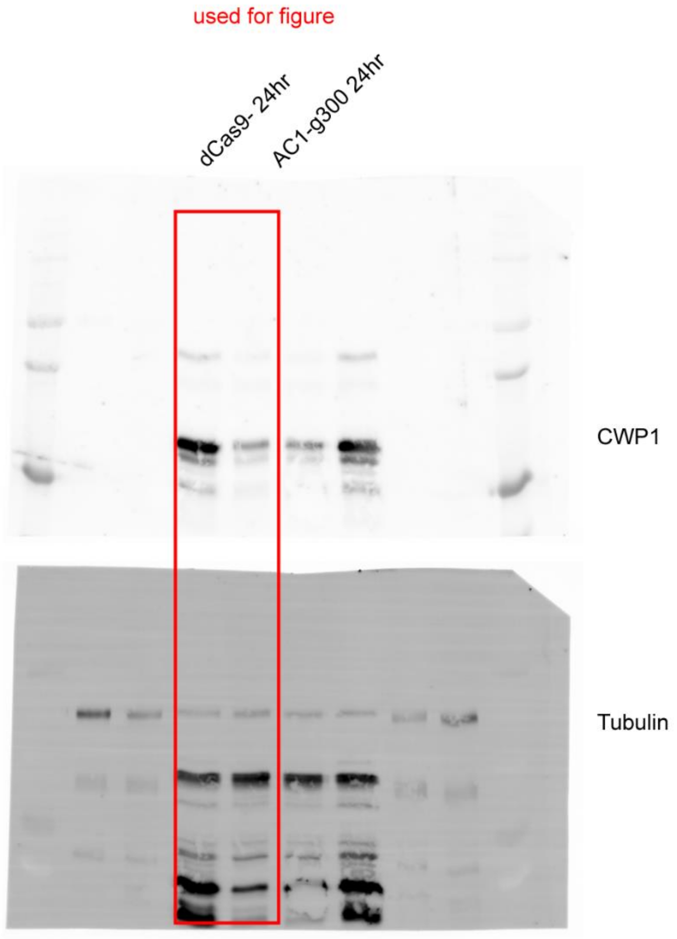

Supplementary Fig4g Western blot: TET inducibled mNG-AC1 Encysted

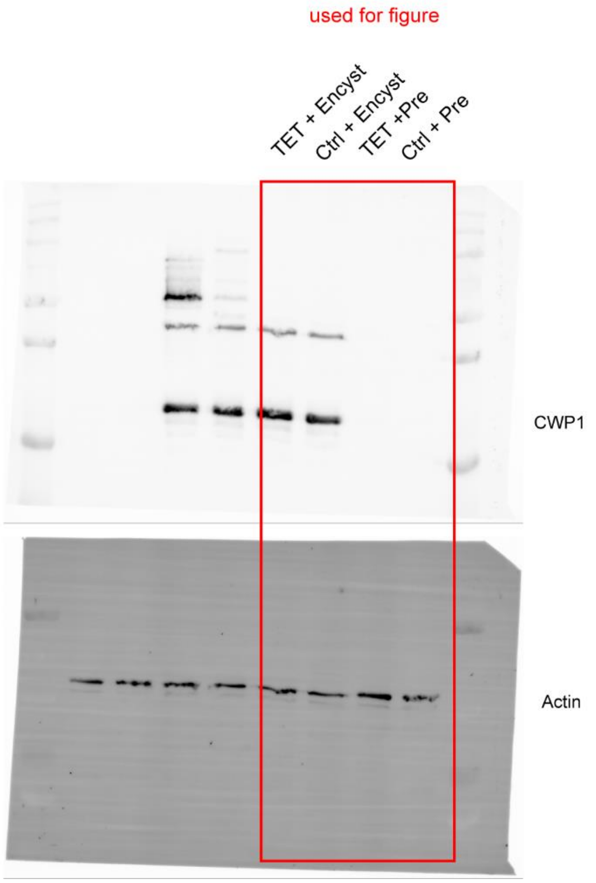

Supplementary Fig8a Western blot: SQ22536 treated Encysted

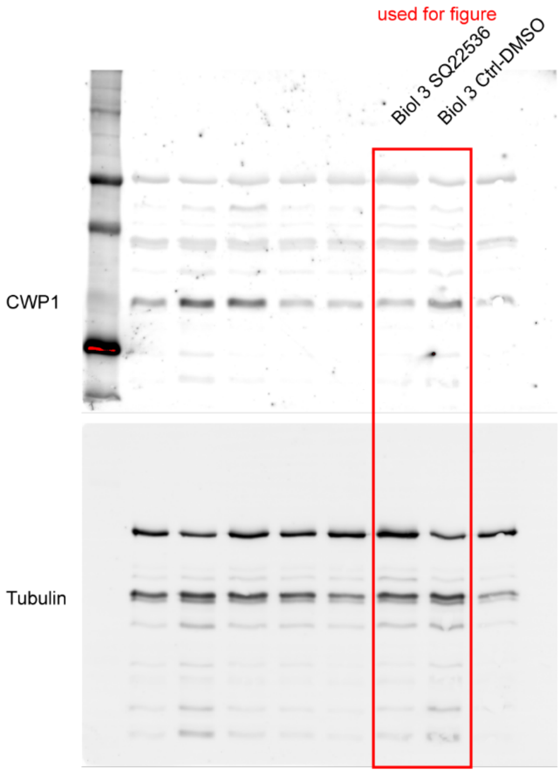

Supplement: Supplementary file 6 — Source Data [file 41467_2023_43028_MOESM6_ESM.zip › Uncropped blots.pdf]
